# Prediction of acid radical ion binding residues by K-nearest neighbors classifier

**DOI:** 10.1186/s12860-019-0238-8

**Published:** 2019-12-11

**Authors:** Liu Liu, Xiuzhen Hu, Zhenxing Feng, Xiaojin Zhang, Shan Wang, Shuang Xu, Kai Sun

**Affiliations:** College of Sciences, Inner Mongolia University of Technology, Hohhot, 010051 China

**Keywords:** K-nearest neighbors classifier, Acid radical ions, Binding residues

## Abstract

**Background:**

Proteins perform their functions by interacting with acid radical ions. Recently, it was a challenging work to precisely predict the binding residues of acid radical ion ligands in the research field of molecular drug design.

**Results:**

In this study, we proposed an improved method to predict the acid radical ion binding residues by using K-nearest Neighbors classifier. Meanwhile, we constructed datasets of four acid radical ion ligand (NO_2_^−^, CO_3_^2−^, SO_4_^2−^, PO_4_^3−^) binding residues from BioLip database. Then, based on the optimal window length for each acid radical ion ligand, we refined composition information and position conservative information and extracted them as feature parameters for K-nearest Neighbors classifier. In the results of 5-fold cross-validation, the Matthew’s correlation coefficient was higher than 0.45, the values of accuracy, sensitivity and specificity were all higher than 69.2%, and the false positive rate was lower than 30.8%. Further, we also performed an independent test to test the practicability of the proposed method. In the obtained results, the sensitivity was higher than 40.9%, the values of accuracy and specificity were higher than 84.2%, the Matthew’s correlation coefficient was higher than 0.116, and the false positive rate was lower than 15.4%. Finally, we identified binding residues of the six metal ion ligands. In the predicted results, the values of accuracy, sensitivity and specificity were all higher than 77.6%, the Matthew’s correlation coefficient was higher than 0.6, and the false positive rate was lower than 19.6%.

**Conclusions:**

Taken together, the good results of our prediction method added new insights in the prediction of the binding residues of acid radical ion ligands.

## Introduction

The protein is the foundation of life and participates in almost all life processes, such as heredity, growth and development. Most of the proteins need to binding with other specific proteins and form a protein complex to perform their normal biological functions, and previous researchers have made numerous related works and gave us more understanding for the mechanism proteins functions [1–10]. Many proteins require binding to acid radical ions to perform their functions. For instance, protein enzymes bind to phosphate ions (PO_4_^3−^), which cause phosphorylation that can regulate enzyme activity [11]; sulfate ion are involved in several important processes of cell metabolism, such as the synthesis process of cysteine and the sulfation process of protein [12, 13]. However, it’s still a limit to completely understand the cellular mechanism of protein function. Therefore, it is a valuable work to accurately predict the binding residues of acid radical ion ligands, which can help us illustrate the function of proteins.

Up to now, some researchers have studied acid radical ion binding residues by the experimental methods. In 1966, Pardee used the experimental method to study proteins combining with sulfate ion in Salmonella typhimurium, and analyzed the mechanism of interaction between sulfate ion and binding residues [14]. In 2002, the experimental method was adopted by Richard et al. to study the interaction between proteoglycans and sulfate ions, locating the sites of interaction with heparan sulfate in the protein [15]. Tamada studied the sulfation of proteins by the experimental method in 2003 [16]. Some researchers have studied acid radical ion binding residues by the theoretical methods. For instance, Hu et al. developed the model (IonSeq) for predicting four acid radical ion (NO_2_^−^, CO_3_^2−^, SO_4_^2−^, PO_4_^3−^) binding residues that were taken from the BioLip database and achieved an accuracy of nearly 98% for all ions in 2016 [17]. In 2016, Hu et al. predicted binding residues of SO_4_^2−^ and PO_4_^3−^ in the BioLip database by the ensemble classifier, and obtained Matthew’s correlation coefficient was higher than 0.23 and overall accuracy was higher than 97% [18]. In 2017, SVM algorithm was used by Li et al. to identify the binding residues of SO_4_^2−^ from LPC, achieving the Matthew’s correlation coefficient of 0.571 and the overall accuracy of 78.5% in the five-fold cross-validation [19]. In 2017, Zhang et al. updated the online server COFACTOR by combining structure, sequence and protein-protein interaction information to improve proteins function prediction, in which obtained the Matthew’s correlation coefficient was greater than that obtained by Concavity and Findsite for ligand-binding residues of the same set of proteins [20]. 2018, Peyton et al. used an interpretable confidence-rated boosting algorithm to predict protein-ligand interactions with high accuracy from ligand chemical substructures and protein sequence motifs [21].

In this paper, we reconstructed datasets of acid radical ion ligands from BioLip database and developed an improved method to predict the binding residues of four acid radical ions. We explored the optimal window length and extracted the refined characteristics from the composition and position information. Besides, we also integrated the information of amino acid, hydrophilic-hydrophobic, polarization charge and predicted structure as characteristics parameters for the K-nearest Neighbors classifier. In comparison with previous work, we obtained better results in the predicted of NO_2_^−^, CO_3_^2−^ and PO_4_^3−^ ligands.

## Materials and methods

### Dataset

BioLip database contains 13 acid radical ion ligands. The proteins interacting with acid radical ions were downloaded from BioLip database and their pairwise sequence identity was below 95%. Then, the proteins with a resolution less than 3 Å and a sequence length above 50 residues were further selected. Finally, the proteins with sequence identity threshold higher than 30% were removed using the CD-HIT software [22]. Through the above screening, it was found that the number of binding residues of only four acid radical ions (NO_2_^−^, CO_3_^2−^, SO_4_^2−^ and PO_4_^3−^) conformed to the requirement of statistical analysis. Other acid radical ion ligands, such as Cl^−^, WO_4_^2−^, NO_3_^−^, SO_3_^2−^, PO_3_^3−^ have fewer number of binding residues. Therefore, we only selected the binding residues of NO_2_^−^, CO_3_^2−^, SO_4_^2−^ and PO_4_^3−^ as research objects. The non-redundant datasets of the four acid radical ion ligands were shown in Table 1.

**Table 1:** Benchmark dataset of four acid radical ions

Acid radical ion	Chains	Positive segments	Negative segments
NO_2_^−^	22	98	8144
CO_3_^2−^	62	316	22,766
SO_4_^2−^	303	2125	99,729
PO_4_^3−^	339	2168	112,279

Since the interaction between the proteins and ligands is not only related to the binding residues, but surrounding residues also have certain effects, we used the sliding window method to cut the protein sequence into overlapping segments according to the window length of 5, 7, 9, 11, 13, 15 and 17 amino acid residues, respectively. If the central residue was an acid radical ion binding residue, we grouped it into positive; otherwise, we grouped it into negative segment. In order to ensure that each amino acid residue appeared in the center of the segment, we added a (L-1)/2 dummy residue “X” at both terminals of the protein chains, where L is the length of the amino acid sequence segments.

### The selection of feature parameters

Based on our group’s previous research on acid radical ion binding residues [19], it was found that the information of amino acid, polarization charge, hydrophilic-hydrophobic, predicted secondary structures and relative solvent availability could well identify the binding residues of acid radical ion ligands. Therefore, we selected the information of these five basic parameters to predict binding residues of four acid radical ion ligands.

The polarization charge, hydrophilic-hydrophobic, and relative solvent accessibility have different classifications according to various standards. The twenty amino acids are divided into three categories according to the polarization charge, including positively charged amino acids (K, R, P), negatively charged amino acids (D, E), and uncharged amino acids (N, Q, H, L, I, V, A, M, F, S, T, Y, W, C, G) [23]; they are grouped into six categories according to the hydrophilic-hydrophobic properties (Table 2) [24]. In this paper, the relative solvent accessibility (RSA) threshold value of 25% is chosen to indicate whether the residue is exposed (RSA > 25%) or buried (RSA < 25%).

**Table 2:** Hydrophilic-hydrophobic classification of amino acids

Classification	Amino Acids	Classification	Amino Acids
strongly hydrophobic	R, D, E, N, Q, K, H	Proline	P
strongly hydrophilic	L, I, V, A, M, F	Glycine	G
weakly hydrophilic	S, T, Y, W	Cysteine	C

There are three predicted secondary structures: *α*-helix (H), *β*-strand (E) and coil (C).

### Extraction methods of feature parameters

#### Increment of diversity algorithm

Increment of diversity (ID) algorithm is of great significance to the research of biology. It has achieved success in the prediction of subcellular localization and protein folds [25, 26]. It not only can be used as an algorithm for prediction, but also can reduce dimension and refine composition information into discrete increment (ID) values. Its use in this paper belonged to the latter. ID algorithm is introduced as follows:

In the state space of dimension S, the measure of diversity for a vector X: [n_1_, n_2_, …, n_s_] is

1 $$ D(X)=N\log N-\sum \limits_{i=1}^s{n}_i\log {n}_i $$

In the two state spaces of dimension S, for two vectors X: [n_1_, n_2_, …, n_s_] and Y: [m_1_, m_2_, …, m_s_], the measure of diversity for mixed diversity resources X + Y is

2 $$ D\left(X,Y\right)=\left(N+M\right)\log \left(N+M\right)-\sum \limits_{i=1}^s\left({n}_i+{m}_i\right)\log \left({n}_i+{m}_i\right) $$

Here, $N=\sum \limits_{i=1}^s{n}_i\log {n}_i$ , $\mathrm{M}=\sum \limits_{i=1}^s{m}_i\log {m}_i$. *n*_*i*_ / *m*_*i*_ is the number of occurrences of *i*^*th*^ information symbol in the state space.

The increment of diversity of X and Y is

3 $$ ID\left(X,Y\right)=D\left(X+Y\right)-D(X)-D(Y) $$

The measure of diversity is a measure of information diversity, it can describe the uncertainty of the overall information, while the ID is a measure of the spatial similarity between two diversity sources. If X is similar to Y, the value of ID(X, Y) will be small; otherwise, the value of ID(X, Y) will be large. For example, amino acid composition information was input to the ID algorithm, two standard discrete sources were constructed by training set. Then we obtained two ID values for each segment of the testing set. Thus, 20-dimensional vector corresponding to frequencies of 20 amino acids of each sequence segment was compressed into two dimensions. Finally, two ID values were used as feature parameters of the K-nearest Neighbors classifier.

### Position weight scoring matrix

Position weight scoring matrix (PWSM) is a classifier that has achieved great success in the prediction of super-secondary structures and transcription factor binding sites in genomes [27, 28]. The PWSM algorithm was used in this paper to extract feature parameters. The scoring function can be defined as:

4 $$ S=\frac{\sum \limits_{i=1}^L{C}_i\left({m}_{i,j}-{m}_{i,\min}\right)}{\sum \limits_{i=1}^L{C}_i\left({m}_{i,\max }-{m}_{i,\min}\right)} $$

Here,

5 $$ {m}_{i,j}=\log \left(\frac{p_{i,j}}{p_{o,j}}\right) $$

${p}_{i,j}=\frac{\left({n}_{i,j}+\frac{\sqrt{N_i}}{21}\right)}{\left({N}_i+\sqrt{N_i}\right)}$, the conserved parameters of i^th^ position is

6 $$ {C}_i=\frac{100}{\log 21}\left(\sum \limits_{i=1}^{21}{p}_{i,j}\log {p}_{i,j}+\log 21\right) $$

Here j is 20 amino acids and dummy residue “X”. *m*_*i*, *j*_ is the matrix element of position weight matrix and denotes the weight probability of the *j*^*th*^ amino acid at the *i*^*th*^ position, *m*_*i*, max_ and *m*_*i*, min_ are the maximum value and minimum value of *m*_*i*, *j*_, respectively. *p*_*i*, *j*_ is the observed probability of the *j*^*th*^ amino acids at the *i*^*th*^ position, and *p*_*o*, *j*_ is the background probability of the *j*^*th*^ amino acid. *n*_*i*, *j*_ is the frequency of *j*^*th*^ amino acids at the *i*^*th*^ position. *N*_*i*_ is total number of amino acids at the *i*^*th*^ position. L is the length of the amino acid sequence segments.

We constructed two standard position weight matrices using binding segments and non-binding segments from training set, respectively. For each segment from the testing set, 2 L-dimensional position information was obtained straightly from standard position weight matrices and two matrix scoring (S) values were obtained by scoring function. Thus, 21*L-dimensional vector corresponding to position conservation of 21 amino acids of each sequence segment was compressed into 2 L and 2 dimensions. We used 2-dimensional S value and 2 L-dimensional position information as feature parameters, respectively.

### Etraction of feature parameters

#### The composition features

Since the same amino acid residue had different frequencies in the binding segments and the non-binding segments, the amino acid composition information was selected as a feature parameter in this paper. We also selected the composition information of polarization charge, hydrophilic-hydrophobic, secondary structure and relative solvent availability as feature parameters.

#### The position features

Since the conservation of amino acids was different at the same position in the binding segments and the non-binding segments, we selected the 2 L-dimensional position amino acid information as a feature parameter, which was obtained from standard position weight matrices. Similarly, we selected the 2 L-dimensional position information of the polarization charge, hydrophilic-hydrophobic, secondary structure and relative solvent availability as feature parameters. Then we combined the position information of five basic parameters as feature parameters, namely position combination features and input to the K-nearest Neighbors classifier to identify binding residues of acid radical ion ligands.

#### The reduced dimension and refined features

For the amino acid composition information, we obtained the 2-dimensional ID value by using the formula (3). We replaced the amino acid composition with the polarization charge composition, hydrophilic-hydrophobic composition, secondary structures composition and relative solvent availability composition and obtained the 2-dimensional ID value, respectively. Therefore, we obtained the 10-dimensional ID value.

For the position amino acid information, we obtained the 2-dimensional S value by using the formula (4). Similarly, for the position information of polarization charge, hydrophilic-hydrophobic, predicted secondary structures and relative solvent availability, we obtained the 2-dimensional S value, respectively. Therefore, we obtained the 10-dimensional S value.

The 10-dimensional ID value and the 10-dimensional S value were combined as feature parameters, namely the 20-dimensional combination feature and input to the K-nearest Neighbors classifier to predict binding residues of acid radical ion ligands.

### K-nearest neighbors classifier

K-nearest Neighbors (KNN) classifier is a statistical-based machine learning method, which was proposed by Cover and Hart in 1967 [29]. The basic idea of KNN classifier is that k nearest samples of a test sample are found by using a distance formula, then the test sample belongs to the category with the largest number in the k nearest samples. Different k values will yield different classification results, the performance of KNN classifier is optimal when k takes an appropriate value. KNN classifier has been widely used in classification and regression problems, and made a great success in predicting various attributes of proteins, such as proteins subcellular localization and protein structure classification [30, 31].

KNN classifier can get better prediction results when it classifies the dataset with small samples, and the predicted results are more accurate when the number of positive and negative samples of dataset is equal. In this paper, the number of samples used was not large, and negative samples with the equal number of positive samples were randomly sampled. These characteristics matched up the KNN classifier model. Therefore, we used the KNN classifier to identify the four acid radical ion ligand binding residues. Since the algorithm was very mature, we adopted KNN classifier on the weka3.8 platform. The distance formula chosen was Euclidean distance [32–34].

### The validation and evaluation metrics

The proposed method was evaluated by the five-fold cross-validation. The dataset was randomly divided into five equal parts. Four parts were used for training, and remaining one part was used for testing. This process was repeated 5 times, and each part was used once for testing. The average value of five experimental results was taken as the final result. Because the number of negative samples is much larger than that of positive samples, to ensure the stability of the result, negative samples with equal numbers of positive samples were randomly sampled 10 times. The final result was the average value of the 10 results obtained by the five-fold cross-validation.

The following five measures were used to evaluate the prediction performance of acid radical ion binding residues: sensitivity (Sn), specificity (Sp), accuracy (Acc), Matthew’s correlation coefficient (MCC) and false positive rate (FPR). These measures were defined as:

7 $$ {S}_n=\frac{TP}{TP+ FN}\times 100\% $$

8 $$ {S}_p=\frac{TN}{TN+ FP}\times 100\% $$

9 $$ Acc=\frac{TP+ TN}{TP+ TN+ FP+ FN}\times 100\% $$

10 $$ MCC=\frac{\left( TP\times TN\right)-\left( FP\times FN\right)}{\sqrt{\left( TP+ FP\right)\left( TP+ FN\right)\left( TN+ FP\right)\left( TN+ FN\right)}} $$

11 $$ FPR=\frac{FP}{TN+ FP} $$

Where TP is the number of correctly predicted acid radical ion binding residues, TN is the number of correctly predicted non-binding residues, FP is the number of non-binding residues predicted as binding residues, and FN is the number of binding residues predicted as non-binding residues.

## Results and discussion

### The selection of the optimal k value

For each experiment, the best performance obtained by the five-fold cross-validation is achieved with an optimal k value. The average of optimal k values given by 10 experiments is defined as the optimal k value in the determined window length, and it will be reused in 10 experiments to achieve prediction results. At the window length of 13, taking the selection of optimal k value of position combination features of SO_4_^2−^ ligand as an example, we elaborated on the selection method of the optimal k value.

**Fig. 1:**
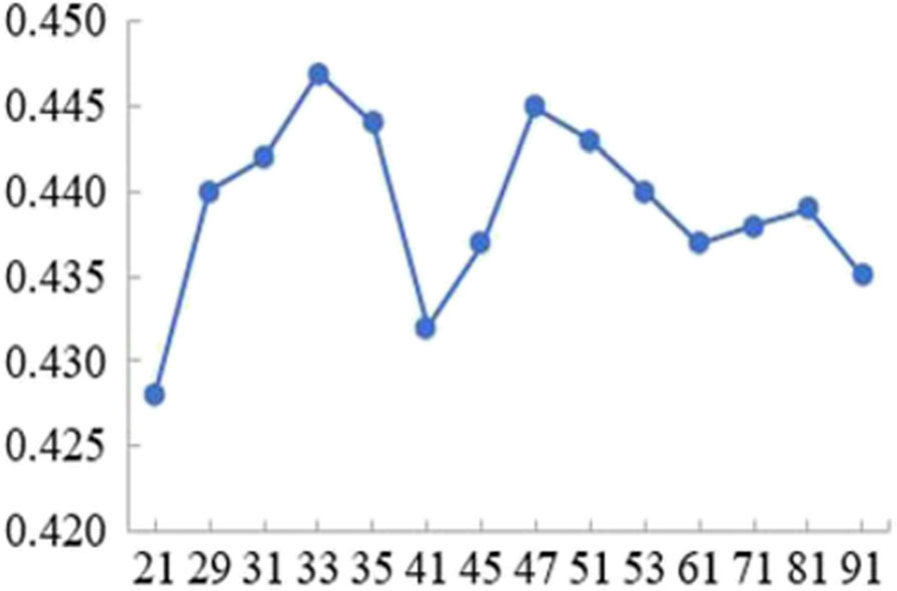
The relation between the values of k and MCC for SO_4_^2−^

Since the negative samples were randomly sampled 10 times, we obtained 10 sample sets. When the window length of SO_4_^2−^ ligand was selected 13, we performed the experiments for ten sample sets respectively. Since different k values would obtain different predicted results, for each experiment, the position combination features were input to the KNN classifier to select optimal k value. For one of the experiments, the obtained relation between k values and the corresponding MCC values was shown in Fig. 1. In Fig. 1, the x-axis represents k value and the y-axis represents the MCC value. As seen, the highest MCC value was 0.447 and the corresponding k value was 33. Therefore, the optimal k value corresponding to the position combination features was 33 in this experiment.

We performed the experiments for other nine sample sets by the same method and the obtained nine optimal k values were 27, 29, 31, 31, 33, 35, 35, 37, 39, respectively. The obtained average value of ten optimal k values was 33. Therefore, at the window length of 13, 33 was the optimal k value which was selected for the position combination features of SO_4_^2−^ ligand.

### The selection of the optimal window length

In the five-fold cross-validation, position combination information was input to the KNN classifier as a characteristic parameter to select the optimal window length (L) of the sequence segments for each acid radical ion. For each window length, based on the position combination features, we selected the optimal k value for them and input them to the KNN classifier to perform 10 experiments with the optimal k value. The average of results obtained by 10 experiments was the final results at each window length. Therefore, we obtained seven results at window length of 5, 7, 9, 11, 13, 15, 17. The window length corresponding to the highest result of the seven results was the optimal window length. Taking the selection of optimal window length of PO_4_^3−^ ligand as an example, we illustrated the method of the selection of the optimal window length.

**Fig. 2:**
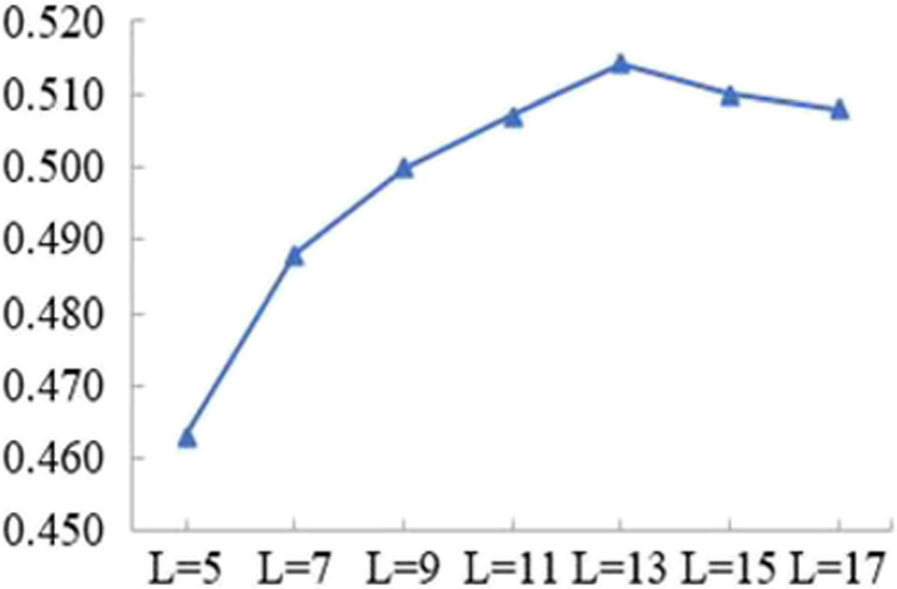
The MCC values of different L

For PO_4_^3−^ ligand, based on the position combination features, we selected the optimal k value for them at each window length. For each window length, we performed experiments using the optimal k value for ten sample sets respectively, and obtained final predicted results. The obtained results by the five-fold cross-validation were given in Fig. 2 and Table 3, respectively.

**Table 3:** Evaluation metrics of position combination features at different L of PO_4_^3−^

L	Optimal k value	Sn (%)	Sp (%)	Acc (%)	FPR (%)	MCC
5	77	76.6	69.6	73.1	30.4	0.463
7	23	76.9	71.8	74.4	28.2	0.488
9	33	76.1	73.8	75.0	26.2	0.500
11	17	76.2	74.5	75.3	25.5	0.507
13	15	76.0	75.4	75.7	24.6	0.514
15	21	76.7	74.4	75.5	25.6	0.510
17	21	78.0	72.8	75.4	27.2	0.508

As shown in Fig. 2, the MCC value of PO_4_^3−^ ligand was increased from window length of 5 to 13. It showed a decreasing tendency from window length of 13 to 17. At window length of 13, PO_4_^3−^ ligand obtained the highest MCC value. At the same time, the values of Sp, Acc and FPR were the highest and the Sn value was approximately equal to that of others when the window length was 13 (see Table 3). Therefore, the optimal window length of PO_4_^3−^ ligand was 13.

The optimal window lengths of the other three acid radical ion ligands were selected by the same way. The selected optimal window lengths of NO_2_^−^, CO_3_^2−^ and SO_4_^2−^ were 13, 15 and 13, respectively.

### Predicted results of composition features

The amino acid composition information was input to the KNN classifier as a feature parameter to predict binding residues of the four acid radical ion ligands. The predicted results of the five-fold cross-validation were shown in Table 4.

**Table 4:** The performance of amino acid composition feature by KNN classifier

Ligand	Optimal k value	Sn (%)	Sp (%)	Acc (%)	FPR (%)	MCC
NO_2_^−^	23	54.1	72.4	63.3	27.6	0.270
CO_3_^2−^	37	63.9	52.8	58.4	47.2	0.169
SO_4_^2−^	91	59.3	61.1	60.2	38.9	0.204
PO_4_^3−^	41	62.9	62.1	62.5	37.9	0.250

As shown in Table 4, the Acc values were lower than 65%, and the MCC values were lower than 0.3 for four acid radical ion ligands. Especially for CO_3_^2−^ ligand, the result was the lowest, with the MCC value of 0.169 and the FPR value of 47.2%. The reason may be that the extracted information was incomplete, so we added the composition information of polarization charge, hydrophilic-hydrophobic, secondary structure and relative solvent accessibility for further prediction. The prediction results of the five-fold cross-validation were shown in Table 5. As seen, the performance was improved after adding other composition features. For example, the MCC value was significantly improved from 0.250 to 0.355, the Sn value was increased from 62.9 to 69.1%, and the Acc value was increased by 5.2% for PO_4_^3−^ ligand. It indicates that the newly added features contained valid information, which has great significance for identifying acid radical ion binding residues.

**Table 5:** The performance of composition combination features by KNN classifier

Ligand	Optimal k value	Sn (%)	Sp (%)	Acc (%)	FPR (%)	MCC
NO_2_^−^	77	57.1	76.5	66.8	23.5	0.343
CO_3_^2−^	35	63.6	59.5	61.6	40.5	0.231
SO_4_^2−^	25	66.0	61.7	63.9	38.3	0.277
PO_4_^3−^	71	69.1	66.3	67.7	33.7	0.355

### Predicted results of position combination features

Since the predicted results of the composition features were not good enough, position combination information of amino acid, polarization charge, hydrophilic-hydrophobic, secondary structure and relative solvent accessibility were used as characteristic parameters to recognize four acid radical ion binding residues by KNN classifier. Obtained results of the five-fold cross-validation were shown in Table 6.

**Table 6:** The performance of position combination features by KNN classifier

Ligand	Optimal k value	Sn (%)	Sp (%)	Acc (%)	FPR (%)	MCC
NO_2_^−^	75	81.6	61.2	71.4	38.8	0.438
CO_3_^2−^	31	75.6	67.7	71.7	32.3	0.435
SO_4_^2−^	33	73.5	71.2	72.3	28.8	0.447
PO_4_^3−^	15	76.0	75.4	75.7	24.6	0.514

As seen, the result of PO_4_^3−^ ligand was the highest, the MCC value was 0.514, and the values of Acc, Sn and Sp were all higher than 75%. PO_4_^3−^ ligand was sensitive to the position combination features and could be well identified by position features. However, the predicted results of NO_2_^−^, CO_3_^2−^ and SO_4_^2−^ were less accurate, in which the MCC values were lower than 0.5 and the Acc values were lower than 73%. Probably because these acid radical ion binding residues were less sensitive to position information.

### Predicted results of reduced dimension and refined features

Since predicted results of binding residues of NO_2_^−^, CO_3_^2−^ and SO_4_^2−^ were still lower, the 20-dimensional combination feature was input to the KNN classifier to predict binding residues of acid radical ion ligands. The predicted results of the five-fold cross-validation were given in Table 7.

**Table 7:** Comparison of prediction results of three features

Ligand	Feature	Optimal k value	Sn (%)	Sp (%)	Acc (%)	FPR (%)	MCC
NO_2_^−^	C	77	57.1	76.5	66.8	23.5	0.343
	P	75	81.6	61.2	71.4	38.8	0.438
	R	75	81.6	79.6	80.6	20.4	0.612
CO_3_^2−^	C	35	63.6	59.5	61.6	40.5	0.231
	P	31	75.6	67.7	71.7	32.3	0.435
	R	115	74.4	78.5	76.4	21.5	0.529
SO_4_^2−^	C	25	66.0	61.7	63.9	38.3	0.277
	P	33	73.5	71.2	72.3	28.8	0.447
	R	37	75.8	69.2	72.5	30.8	0.450
PO_4_^3−^	C	71	69.1	66.3	67.7	33.7	0.355
	P	15	76.0	75.4	75.7	24.6	0.514
	R	61	76.4	74.0	75.2	26.0	0.504

As seen, the 20-dimensional combination feature obtained the better results. The values of Acc and Sn were higher than 70%, and the MCC values were higher than 0.45 for four acid radical ions. There are two possible reasons for it. One is that there is complementarity between the composition information and the position information. The other is that the 20-dimensional combination feature does not have redundant information.

### The comparison of predicted results among three kinds of features

To make the comparison of predicted results among composition combination features (C), position combination features (P) and 20-dimensional combination feature (R) more obvious, their results of the five-fold cross-validation obtained by KNN classifier were listed in Table 7.

Compared with the results obtained by composition combination features, the values of MCC, Acc and Sp were increased by using position combination features for all acid radical ions. For example, the MCC value of CO_3_^2−^ ligand was apparently increased from 0.231 to 0.435, the Sn value of NO_2_^−^ ligand was obviously increased from 57.1 to 81.6%. It shows that position combination features contain more valid information.

Compared with the results obtained by position combination features, the predicted results of the 20-dimensional combination feature were better. The predicted results of NO_2_^−^ and CO_3_^2−^ ligands were significantly improved. In terms of NO_2_^−^ ligand, the MCC value was obviously improved from 0.438 to 0.612, the Acc value was increased from 71.4 to 80.6%, and the values of Sn and Sp were balanced between 81.6 and 79.6%, respectively. For CO_3_^2−^ ligand, the FPR value was decreased from 32.3 to 21.5%, the values of Acc and MCC were increased by 4.7 and 9.4%, respectively. As for the SO_4_^2−^ ligand, the MCC value was improved from 0.447 to 0.450 with almost no change. For PO_4_^3−^ ligand, although the values of Acc, MCC and Sp were slightly declined, the Sn value was slightly increased. It may be that 20-dimensional combination feature lost some valid information, resulting in decrease of negative sample identification results.

In the above predicted results, the identification results corresponding to the 20-dimensional combination feature were the best. Therefore, we should select the feature parameters which contain more valid information and input them to the KNN classifier to accurately recognize binding residues of acid radical ion ligands.

### Predicted results of independent test

To evaluate the practicability of KNN classifier, we have made the independent test for four acid radical ion ligand binding residues.

The dataset of four acid radical ion binding residues was divided into two parts, including training dataset that was used to train model and the independent test dataset that was used to test model. The protein chains in training dataset accounted for 80% of the total data. The data of the two datasets was shown in Table 8.

**Table 8:** The data of the training dataset and independent test dataset

Ligand	Training dataset			Independent test dataset		
	Chains	P^a^	N^b^	Chains	P^a^	N^b^
NO_2_^−^	17	76	6218	5	22	1926
CO_3_^2−^	49	252	18,066	13	64	4700
SO_4_^2−^	242	1751	79,164	61	374	20,565
PO_4_^3−^	271	1730	90,786	68	438	21,493

^a^The number of positive (binding) samples

^b^The number of negative (non-binding) samples

In the independent test, we used the optimal window length of each acid radical ion was taken from Section 3.2. Since acid radical ion ligands were sensitive to the 20-dimensional combination feature, we extracted it as feature parameter of independent testing and input it to the KNN classifier to identify binding residues of acid radical ion ligands, in which selected optimal k values were same as Section 3.5. The obtained results were given in Table 9. Besides, the obtained results were compared with that by IonSeq. The results of IonSeq method were taken from literature [17] in which it was obtained by cross-validation.

**Table 9:** Comparison of our independent test with IonSeq

Ligand	Method	L	Optimal k value	Sn (%)	Sp (%)	Acc (%)	FPR(%)	MCC
NO_2_^−^	IonSeq	11	–	18.00	99.78	98.79	–	0.2847
	OUR’S	13	75	40.90	98.60	97.90	1.40	0.3100
CO_3_^2−^	IonSeq	13	–	10.62	99.82	98.58	–	0.2127
	OUR’S	15	115	48.40	95.00	94.40	5.00	0.2170
SO_4_^2−^	IonSeq	11	–	13.65	99.32	97.53	–	0.1906
	OUR’S	13	37	43.90	86.80	85.80	13.20	0.1160
PO_4_^3−^	IonSeq	11	–	24.15	99.38	97.95	–	0.3121
	OUR’S	13	61	63.20	84.60	84.20	15.40	0.1810

As seen from Table 9, the Sn values obtained by KNN classifier were all higher than those obtained by IonSeq for all acid radical ions. The MCC values of NO_2_^−^ and CO_3_^2−^ ligands by KNN classifier were slightly higher than that by IonSeq, while the MCC values of SO_4_^2−^ and PO_4_^3−^ ligands were lower than that by IonSeq. There are three possible reasons for it. First, the datasets used are different for two models. IonSeq model is aimed at imbalanced dataset, while model constructed in this paper is aimed at dataset with equal number of positive and negative samples. Second, the model in this paper is tested by independent test, but the IonSeq model is tested by cross-validation. Third, the feature parameters used are different. Both methods have their own advantages and can only be roughly compared.

### Predicted results of metal ion ligand binding residues

In order to test the reliability of proposed method, we used the KNN classifier to predict binding residues of the six kinds of metal ion (Zn^2+^, Fe^2+^, Fe^3+^, Cu^2+^, Mn^2+^, Co^2+^) with more binding proteins, and compared the results with that obtained by SVM used in literature [35]. Dataset of six kinds of metal ion binding residues used in this paper was taken directly from literature [35] (Table 10). Since the predicted results of acid radical ion binding residues were higher at the 20-dimensional combination feature, it was also extracted as feature parameter and input to the KNN classifier to predict binding residues of six metal ions. The obtained results of the five-fold cross-validation were given in Table 11.

**Table 10:** Benchmark dataset of six metal ion ligands

Metal ions	Chains	Binding residues	Non-binding residues
Zn^2+^	1428	6408	405,113
Fe^2+^	92	382	29,345
Fe^3+^	217	1057	68,829
Cu^2+^	117	485	33,948
Mn^2+^	459	2124	156,625
Co^2+^	194	875	55,050

**Table 11:** Comparison of results between KNN classifier with SVM

Ligand	Method	L	Optimal k value	Sn (%)	Sp (%)	Acc (%)	FPR (%)	MCC
Zn^2+^	OUR’S	7	103	94.3	83.8	89.1	16.2	0.786
	SVM		–	99.8	99.5	99.7	–	0.993
Fe^2+^	OUR’S	9	41	92.1	80.4	86.3	19.6	0.730
	SVM		–	91.9	90.7	91.3	–	0.826
Fe^3+^	OUR’S	9	15	84.6	84.9	84.7	15.1	0.694
	SVM		–	86.9	88.7	87.8	–	0.756
Cu^2+^	OUR’S	13	49	92.4	86.6	89.5	13.4	0.791
	SVM		–	95.5	97.1	96.3	–	0.926
Mn^2+^	OUR’S	7	23	79.1	80.9	80.0	19.1	0.600
	SVM		–	82.1	84.4	83.2	–	0.664
Co^2+^	OUR’S	11	99	77.6	83.1	80.3	16.9	0.608
	SVM		–	80.8	85.1	83.0	–	0.660

Table 11 shows the prediction results of 6 kinds of metal ion ligand binding residues obtained by KNN classifier. The MCC values were higher than 0.6, the values of Acc and Sp were higher than 80%, and FPR percentages were lower than 20%. Predicted results of Zn^2+^ and Cu^2+^ by KNN classifier were relatively better.

Although the predicted results of six metal ions obtained by KNN classifier were lower than that by SVM, their predicted trends were consistent [35]. To achieve better results, the KNN classifier only need to select an optimal k value, while the SVM need to select a group (c, g) optimal values. KNN classifier can achieve similar prediction results with SVM by simpler calculation. The training time complexity of KNN classifier is lower than that of SVM algorithm. The KNN classifier mainly relies on the surrounding limited adjacent samples rather than the method of discriminating class domain to determine the category, and new data can be added directly to the dataset without retraining. KNN classifier theory is simple and easy to implement. Therefore, KNN classifier can be used as auxiliary tool for predicting acid radical ion ligand binding residues.

## Conclusion

To perform the normal biological functions, many proteins require bind to the specific acid radical ions [11–13]. In order to illustrate the proteins function, it is a valuable work to predict the binding residues of acid radical ion ligands in recent years. In this work, we proposed K-nearest Neighbors classifier to predict four acid radical ion ligands binding residues. Firstly, the dataset of acid radical ion ligands was constructed. Then we selected the optimal window length for each acid radical ion ligand. Next, we extracted the composition features, position features and reduced dimension and refined features at the optimal window length, and selected the optimal k value for three feature parameters. The promising results were obtained by K-nearest Neighbors classifier when feature parameters that contained more comprehensive information were used to predict acid radical ion binding residues. In the predicted results, NO_2_^−^, CO_3_^2−^ and PO_4_^3−^ ligands obtained better results by K-nearest Neighbors classifier. For SO_4_^2−^ ligand, other valid information needed to be added in our further work to improve the recognition result.

## Data Availability

The datasets used and analysed during the current study are available from the corresponding author on reasonable request.

## Abbreviations

Acc: Accuracy
FPR: False positive rate
ID: Increment of diversity
KNN: K-nearest Neighbors
LPC: Ligand Protein Contact
MCC: Matthew’s correlation coefficient
PWSF: Position weight scoring matrix
RSA: Relative solvent accessibility
Sn: Sensitivity
Sp: Specificity
SVM: Support vector machine
